# Role of hyaluronic acid in the treatment of peri-implant diseases: results of a meta-analysis

**DOI:** 10.3389/froh.2025.1564599

**Published:** 2025-05-01

**Authors:** Nansi López-Valverde, Antonio López-Valverde, José Antonio Blanco Rueda

**Affiliations:** Department of Surgery, University of Salamanca, Instituto de Investigación Biomédica de Salamanca (IBSAL), Salamanca, Spain

**Keywords:** hyaluronic acid, dental implants, peri-implantitis, clinical trial, meta-analysis

## Abstract

Peri-implantitis is an infectious-inflammatory disease that affects the tissues surrounding implants and is one of the main causes of implant failure. Hyaluronic acid (HA) is a natural polymer with multiple biomedical and cosmetic applications. The aim of this systematic review and meta-analysis was to evaluate its efficacy in the treatment of peri-implant disease.

**Methods:**

In accordance with PRISMA, the question was established: is HA treatment effective as a sole or adjunctive therapy for the treatment of peri-implantitis? PubMed/Medline, Embase, Cochrane Central, Dentistry & Oral Sciences Source and Web of Science were searched until December 2024. Inclusion criteria were interventional studies (RCTs and case series), according to the PICOs strategy in subjects with peri-implant pathology (participants), treated with HA (intervention) compared to conventionally treated or untreated patients (control) and assessing response to treatment (outcomes).

**Results:**

Thirty-two studies were obtained and four were selected. Risk of bias was assessed using the Cochrane Risk of Bias tool and methodological quality using the Joanna Briggs Institute tool. Meta-analysis of parameters was performed for pooled studies and for subgroups. The overall effect was in favour of the experimental group.

**Conclusions:**

The use of HA as background or adjunctive therapy in peri-implantitis may be effective, although well-designed RCTs are warranted to validate the efficacy of the product.

**Systematic Review Registration:**

Identifier (INPLANSY 2024100050).

## 1 Introduction

Dental implants have become a reliable treatment for the replacement of missing teeth, with successful results approaching 83% of the cases treated after 16 years of follow-up (1); although some of them fail in the short, medium or long term. This failure is the result of multiple factors: age, smoking, certain systemic pathologies, the site of placement in the maxilla, the quantity and quality of available bone, etc. However, the most frequent local cause of failure is infection (2). The term peri-implantitis describes an inflammatory response to an infection induced by the accumulation of bacterial plaque on the surface of the implant biomaterial, which leads to a loss of supporting bone, its progression being influenced not only by bacterial dysbiosis but also by the host's inflammatory response (3, 4).

Surgical and non-surgical interventions have been proposed for their treatment, with the aim of reducing the peri-implant pocket and bleeding on probing and, therefore, radiographic consolidation of the peri-implant bone. Non-surgical methods include the use of teflon instruments, titanium tips, ultrasonic tips or laser, however, the rough surfaces of implants are difficult to decontaminate because they favor bacterial adhesion. Because of this, this type of treatment is often used in combination with adjunctive techniques such as antiseptic agents or antibiotics (5). However, the treatment of peri-implantitis has become a challenge, due to the difficulty of adequately decontaminating the implant surface, which is of vital importance for the successful resolution of bone defects created by the disease (6).

Hyaluronic acid (HA) is a natural polymer belonging to the glycosaminoglycan family, which is abundant in the extracellular matrix of periodontal tissues (7). It has several physiological functions capable of regulating osmotic pressure and tissue lubrication, helping to maintain the homeostatic and structural integrity of tissues, making it an ideal biomaterial for medical applications (8). In addition, it can act as an external cytoskeleton, modifying and controlling cell morphology and regulating tissue repair processes by activating inflammatory cells that initiate a response to injury and regulate the behavior of epithelial cells and fibroblasts, thus, it may play an important role in the inflammatory response, as high molecular weight HA degrades to lower molecular weight molecules in inflamed tissues such as in the postoperative period after implant surgery (9, 10). In recent years, HA formulations have been developed for topical administration as adjuvant treatment in acute and chronic gingival diseases and in tissue healing after oral surgery, generally based on preclinical studies (11, 12). However, despite the existence of studies on the role of HA in the field of dentistry, clinical studies evaluating the role of HA in peri-implant tissues are scarce and a standard treatment for peri-implantitis cannot yet be extracted from the clinical literature, although its properties as a mediator of the inflammatory response and regulator of tissue regeneration processes, in addition to its demonstrated role in angiogenesis and neovascularization, make it a suitable biomaterial for the adjuvant treatment of peri-implant lesions (13).

Studies on the efficacy of HA in peri-implant diseases are scarce and, to our knowledge, no literature review has been performed, so the aim of the present systematic and meta-analytic review of randomized clinical studies was to evaluate the efficacy of HA in the treatment of peri-implant diseases.

## 2 Materials and methods

### 2.1 Study presentation and registration

This systematic review and meta-analysis have been prepared according to “The Pre-ferred Reporting Items for Systematic Reviews and Meta-Analyses” (PRISMA) (14) and the Cochrane Handbook guidelines (15). The protocol of this meta-analysis has been registered in INPLASY under the number: INPLASY2024100050, doi number: 10.37766/inplasy2024.10.0050.

### 2.2 Question of interest

The focus of the research question was formulated according to the PICOs format: “Is HA treatment effective as a sole or adjuvant therapy for the treatment of peri-implantitis?”.

Intervention studies in adult patients with peri-implantitis (P) comparing HA treatment (I) with patients receiving conventional treatment or no treatment (C) were included to observe the effects on clinical parameters (O), and only randomized clinical studies were considered (Table 1).

**Table 1:** PICOs format.

Population	Adult subjects with peri-implantitis
Intervention	Treatment with HA alone or in combination with other products
Comparisons	Conventional surgical or non-surgical treatment
Outcomes	Observe the effects of treatment on clinical parameters indicative of peri-implantitis (Δ PD; Δ AL; Δ MBL; Δ BOP)
Study design	Randomized Controlled Trials (RCTs) and case series with at least 10 patients

Δ, variable increase; PD, probing depth; AL, attachment loss; MBL, marginal bone level; BOP, bleeding on probing.

### 2.3 Studies selection; inclusion and exclusion criteria

The original research studies were selected according to the following inclusion criteria: (i) randomized clinical trials (single or double blind) with more than 10 participants aged 18 years or over; (ii) case series with at least 10 patients; (iii) dealing with peri-implant pathologies; (iv) providing data on clinical parameters indicative of peri-implant disease; (v) that used statistical methods including means and standard deviation, together with the units of measurement of mediator levels; (vi) published in English. Studies that did not meet all the criteria, that lacked data on peri-implant disease, experimental studies in animals or *in vitro*, clinical cases or case series with fewer than 10 patients, literature reviews and non-relevant studies (editorials, conference contributions, historical reviews, etc.) were excluded.

### 2.4 Search approach

Two reviewers (NL-V, AL-V) conducted independent searches of the PubMed/Medline, Embase, Cochrane Central, Dentistry & Oral Sciences Source and Web of Science (WOS) databases up to August 2024. They used the Medical Subject Headings (MeSH) terms: Peri-Implantitis*/diagnosis OR Peri-Implantitis*/AND Dental Implants* AND Dental Plaque* AND Hyaluronic Acid/therapeutic use* AND Humans*. In addition, a manual search was carried out and the gray literature was consulted. The bibliographic references of the included studies were also examined to obtain as much information as possible (Table 2).

**Table 2:** Search strategy.

Databases	Search terms
PubMed/Medline	Peri-Implantitis*/diagnosis OR Peri-Implantitis*/AND Dental Implants* AND Dental Plaque* AND Hyaluronic Acid/therapeutic use* AND Humans*
Embase	Peri-Implantitis AND Dental Implants AND Dental Plaque*AND Hyaluronic Acid
Cochrane central	Peri-Implantitis AND Dental Implants AND Hyaluronic Acid
Dentistry & oral sciences	Peri-Implantitis AND Dental Implants AND Hyaluronic Acid
Web of science	Peri-Implantitis AND Dental Implants AND Dental Plaque AND Hyaluronic Acid AND Humans
Boolean operators	AND y OR

### 2.5 Data extraction

Two reviewers (NL-V and AL-V) extracted and tabulated the data from each included study using the standardized JBI-MAStARI data extraction tools. Similarly, the titles and abstracts of the pre-selected studies were reviewed by both reviewers. Those that met the inclusion criteria were read in full and data were extracted. Discrepancies between reviewers were resolved through discussion and the mediation of a third reviewer (JABR). Cohen's kappa index (*κ*) (16) was used to assess inter-rater agreement. Because all the articles included were randomized studies, the data extraction form “The Joanna Briggs Institute Meta-Analysis of Statistics Assessment and Review Instrument (JBI-MAStARI)” specific for randomized controlled trials was used. The data extracted from the studies included specific details of the interventions, study methods, populations, specific objectives and significant results, in order to formulate the question of interest. The results were subjected to double data entry to minimize errors.

### 2.6 Methodological rigor of the studies; evaluation of study quality

The methodological quality of the studies included in the meta-analysis was evaluated with the JBI MAStARI tool. This instrument considers the evidence and the specific methods used in RCTs to synthesize different types of evidence. The checklist consists of thirteen items, with possible answers of “yes”, “no”, “unclear”, or “not applicable”. A “yes” answer scores one point. To be included, a study had to obtain a minimum score of seven (17).

### 2.7 Analysis

The data obtained from the selected RCTs were analyzed with Review Manager software (RevMan Software. Version 5.4.1; The Cochrane Collaboration, Copenhagen, Denmark; 2020). A meta-analysis of the pooled studies and a subgroup analysis were performed for each of the variables that evaluated peri-implantitis. All analyses were based on the mean difference (MD) and standard deviation (SD) to estimate continuous data, and on 95% confidence intervals (CI) to evaluate categorical data. Heterogeneity was considered not important with an I^2^ of 0%–30%; moderate with an I^2^ of 40%–50%; substantial with an I^2^ of 60%–75%; and considerable with an I^2^ ≥ 75%. The threshold for statistical significance was set at *p* < 0.05. Due to the homogeneity of the results, a fixed-effect meta-analysis was carried out.

### 2.8 Risk of bias

Two investigators (NL-V and AL-V) independently assessed the risk of bias of studies using the Cochrane Risk of Bias Tool (RoB2) (18), using 7 domains: Random sequence generation (Selection bias); Allocation concealment (Selection bias); Blinding of participants and personnel (Execution bias); Blinding of outcome assessment (Detection bias); Incomplete outcome data (Attrition bias). Studies were assessed with “high”, “low” and “borderline” risk of bias; “borderline” risk of bias was applied to that lacking information on possible bias. Discrepancies among the evaluators were discussed to reach consensus.

## 3 Results

**Figure 1:**
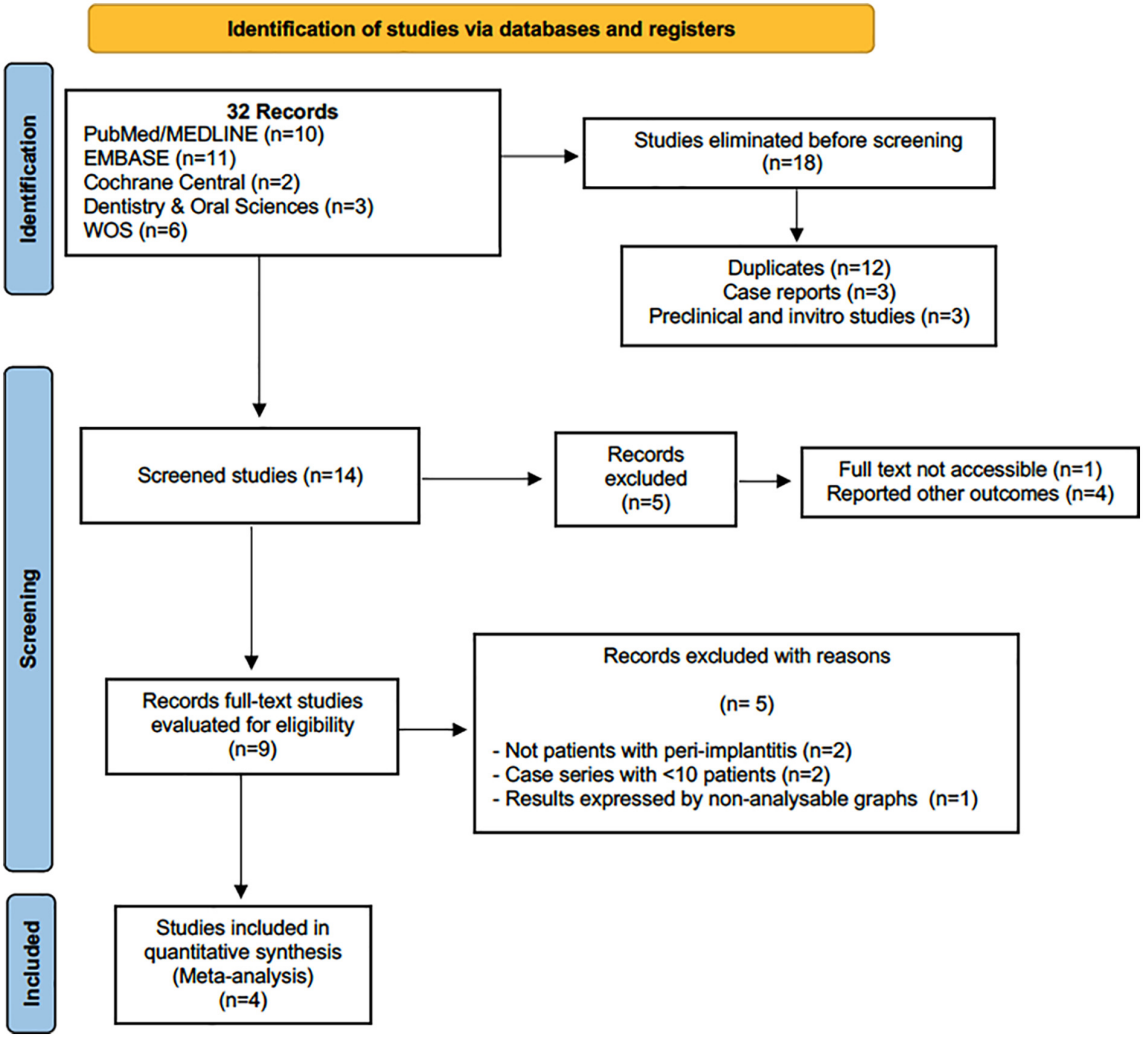
Flowchart.

The electronic search found a total of 32 results of which 9 full-text publications were evaluated and 5 were excluded based on *a priori* criteria, resulting in 4 studies included in the meta-analysis (19–22). Inter-reviewer agreement in including studies exceeded 90% (κ >90%) (Figure 1).

### 3.1 Characteristics of the studies

Thirty-two studies were originally identified, 18 of which were eliminated after the first screening. Subsequently, five more studies were eliminated, and then another five, finally leaving four studies for the meta-analysis (19–22), of which three were RCTs (19, 20) and one (22) was a prospective study. A total of 152 patients and 242 implants were studied. The follow-up periods ranged from 1.5 to 12 months.

The study by Soriano-Lerma et al. (19) evaluated the effects of an HA gel on the microbiome of implants with peri-implantitis in 63 patients and 104 implants after 45 days, reporting a decrease in microbial diversity after treatment with HA, compared to the control group.

Sánchez-Fernández et al. (20) investigated the effects of HA on peri-implant clinical variables and the crevicular concentrations of the pro-inflammatory biomarker's interleukin IL-1β and tumor necrosis factor α (TNF-α) in 63 patients and 104 implants in patients with peri-implantitis, finding, after 90 days, a decrease in bleeding on probing, as well as significantly lower PD values in the test group at 45 and 90 days. Similarly, implants with PD ≥5 mm showed higher levels of IL-1β in the control group at 45 days than in the test group.

Rakašević et al. (21) evaluated the clinical and radiographic efficacy of a bovine bone substitute fused with HA in reconstructive surgery of peri-implant bone defects in 13 patients with 19 implants placed and reported a significantly greater vertical gain in minimum crest height at 6 months in the test group compared to the control group.

The prospective study by Friedmann et al. (22) evaluated the efficacy of a cross-linked collagen matrix biofunctionalized with HA as a reconstructive therapy for peri-implantitis, reporting complete pocket closure in the sixth week after surgery. They also found a significant reduction in the number and frequency of bleeding sites, together with a significant reduction in pocket depth and an increase in mineralized tissue (Tables 3 and 4).

### 3.2 Assessment of methodological rigor

The methodological quality of all included studies ranged from very high (>10 points) to high (10 points), as determined by the JBI- MAStARI critical appraisal checklist for RCTs. The study by Friedmann et al. (22) was not evaluated because it was a prospective study (Table 5).

**Table 3:** Characteristics of studies and participants included in the meta- analysis.

Study, year	Type of study	Subjects number	Implants evaluated	Peri-implantitis criteria	Follow-up	Clinical data analyzed	Detection method	Outcomes
Soriano-Lerma et al. 2019 (19)	A double-blinded, controlled, randomized clinical trial (three parallel groups)	63	104	According to the criteria of the Association of Dental Implantology (PD ≥4 mm, bleeding on probing, and radiological marginal bone loss >2 mm).	45 days	PD, AL, MBL, BOP	Subgingival plaque samples by the Mombelli method (23). DNA isolation was performed according to Lewis et al. (24) with some modifications.	Three strata were obtained from 108 samples with different microbial composition three main microbial consortia associated with peri-implantitis. Stratum 1 showed no differences for any variable after HA treatment, whereas in stratum 2, *Streptococcus*, *Veillonella*, *Rothia* and *Granulicatella* decreased (*P* < 0.05). Similarly, *Prevotella* and *Campylobacter* (*p* < 0.05) decreased in stratum 3 after HA treatment. A decrease in microbial diversity was observed in stratum 3 (*P* < 0.05) after HA treatment compared to the control group.
Sánchez-Fernández et al. 2021. (20)	A double-blinded, controlled, randomized clinical trial (three parallel groups)	63	104	According to the criteria of the Association of Dental Implantology (PD ≥4 mm, bleeding on probing, and radiological marginal bone loss >2 mm).	45, and 90 days.	PD, AL, MBL, BOP	PCPUNC 15 hand-held probe (Hu-Friedy, Chicago, IL, USA) to determine PD at 4 implant sites (mesial, vestibular, distal and palatal/lingual), clinical adhesion loss and peri-implant bleeding. Periapical radiovisiography to determine the MBL. Mombelli technique (19) for crevicular fluid collection	PD was significantly lower in the test group than in control groups at 45 and 90 days. There was a trend toward less bleeding on probing in the test group than in control group 2 at 90 days. Implants with a PD ≥5 mm showed higher IL-1β levels in the control group at 45 days than in the test group (*p* = 0.04).
Rakašević et al. 2023 (21)	Randomized, cross-over, placebo- controlled, double blind trial.	13	19	According to the 2017 Global Workshop Consensus (BOP +, PD ≥6 mm and MB loss ≥3 mm).	6 Months	PD, AL, MBL, BOP	Graduated probe (PCPUNC 15, Hu-Friedy, Chicago, IL, USA) with a force of 0.25 N. Radiographic examinations to evaluate changes in the MBL level around the implant.	Seventy-five percent of the patients and 83% of the implants achieved treatment success after six months. Clinical outcomes improved over time within the groups; however, with no significant differences between the groups. Vertical MBL gain was significantly higher in the test group compared to the control (*p* < 0.05).
Friedmann et al. 2024 (22)	Prospective case series	13	15	NR	12 Months	PD, AL, MBL, BOP	NR	All sites presented with complete closure by week six after surgery. All implants were followed up for at least 12 months. The number of sites with BOP was substantially reduced to 8 out of 79 resulting in a statistically significant reduction in bleeding frequency (*p* < 0.0001). Probing depth assessed at 12 months was statistically significant (*p* < 0.0001). The extent of defect and mineralised tissue gain exhibited a significant reduction (*p* < 0.0001).

MBL, marginal bone level; AL, attachment loss; PD, probing depth; BOP, bleeding on probing.

**Table 4:** Specific and sociodemographic characteristics of the studies.

Study	Age range (year)	Female, *n* %	Associated morbidity	Implant type	Characteristics of the intervention
Soriano-Lerma et al. (19)	Test: 43–81 Control 1: 54–79 Control 2: 29–78	Test: 66.7% Control 1: 57.1% Control 2: 61.9%	Diabetes, *n* (%) Test: 4.8% Control 1: 9.5% Control 2: 9.5%	Tapered Swiss Plus® (Zimmer Dental, Barcelona, Spain)	Application with a syringe of 0.8% HA gel in the peri-implant pocket.
Sánchez-Fernández et al. (20)	Test: 43–81 Control 1: 54–79 Control 2: 29–78	Test: 66.7% Control 1: 57.1% Control 2: 61.9%	Diabetes, *n* (%) Test: 4.8% Control 1: 9.5% Control 2: 9.5% Hypertension, *n* (%) Test: 28.6% Control 1: 10.0% Control 2: 5.0% Osteoporosis, *n* (%) Test: 14.3% Control 1: 20.0% Control 2: 5.0%	Tapered Swiss Plus® (Zimmer Dental, Barcelona, Spain).	NR
Rakašević et al. (21)	Mean age 46.85 ± 9.96	62%	Healthy patients or patients with mild or moderate systemic conditions or diseases that are well controlled	NR	Peri-implant bone defect reconstruction by bovine bone substitute with HA covered by porcine dermal collagen matrix.
Friedmann et al. (22)	31–79	43%	NR	NR	Matrix of cross-linked collagen with HA adapted to the space between the surface of the implant and the bone walls

NR, no report; HA, hyaluronic acid.

**Table 5:** Methodological quality of included studies according to JBI-MAStARI.

Study	Q1	Q2	Q3	Q4	Q5	Q6	Q7	Q8	Q9	Q10	Q11	Q12	Q13	Total score
Soriano-Lerma et al. (19)	1	1	1	1	1	1	1	1	1	1	1	1	1	13
Sánchez-Fernández et al. (20)	1	1	1	1	1		1	1	1	1	1	1	1	13
Rakašević et al. (21)	0	0	1	0	1	0	1	1	1	1	1	1	1	9
Friedmann et al. (22)	–	–	–	–	–	–	–	–	–	–	–	–	–	–

Q1. Was true randomisation used for assigning participants to treatment groups?; Q2. Was allocation to treatment groups concealed?; Q3. Were treatment groups similar at the baseline?; Q4. Were participants blind to treatment assignment?; Q5. Were those delivering treatment blind to treatment assignment?; Q6. Were outcomes assessors blind to treatment assignment?; Q7. Were treatment groups treated identically other than the intervention of interest?; Q8. Was follow-up complete and if not, were differences between groups in terms of their follow- up adequately described and analysed?; Q9. Were participants analysed in the groups to which they were randomized?; Q10. Were outcomes measured in the same way for treatment groups?; Q11. Were outcomes measured in a reliable way?; Q12. Was appropriate statistical analysis used?; Q13. Was the trial design appropriate, and any deviation from the standard RCT design accounted for in the conduct and analysis of the trial?.

### 3.3 Meta-analysis, risk of bias

**Figure 2:**
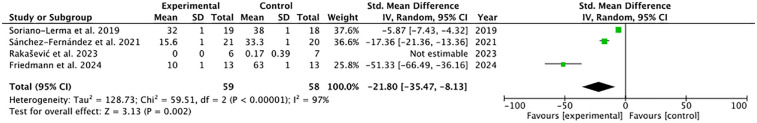
Forest plot of BOP.

**Figure 3:**
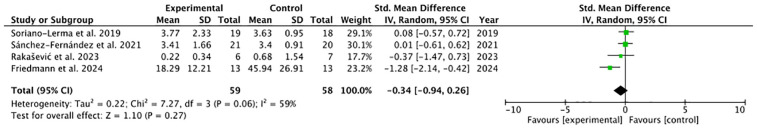
Forest plot of MBL.

**Figure 4:**
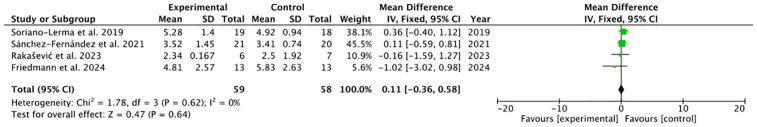
Forest plot AL.

**Figure 5:**
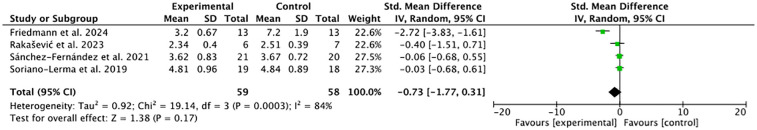
Forest plot of PD.

**Figure 6:**
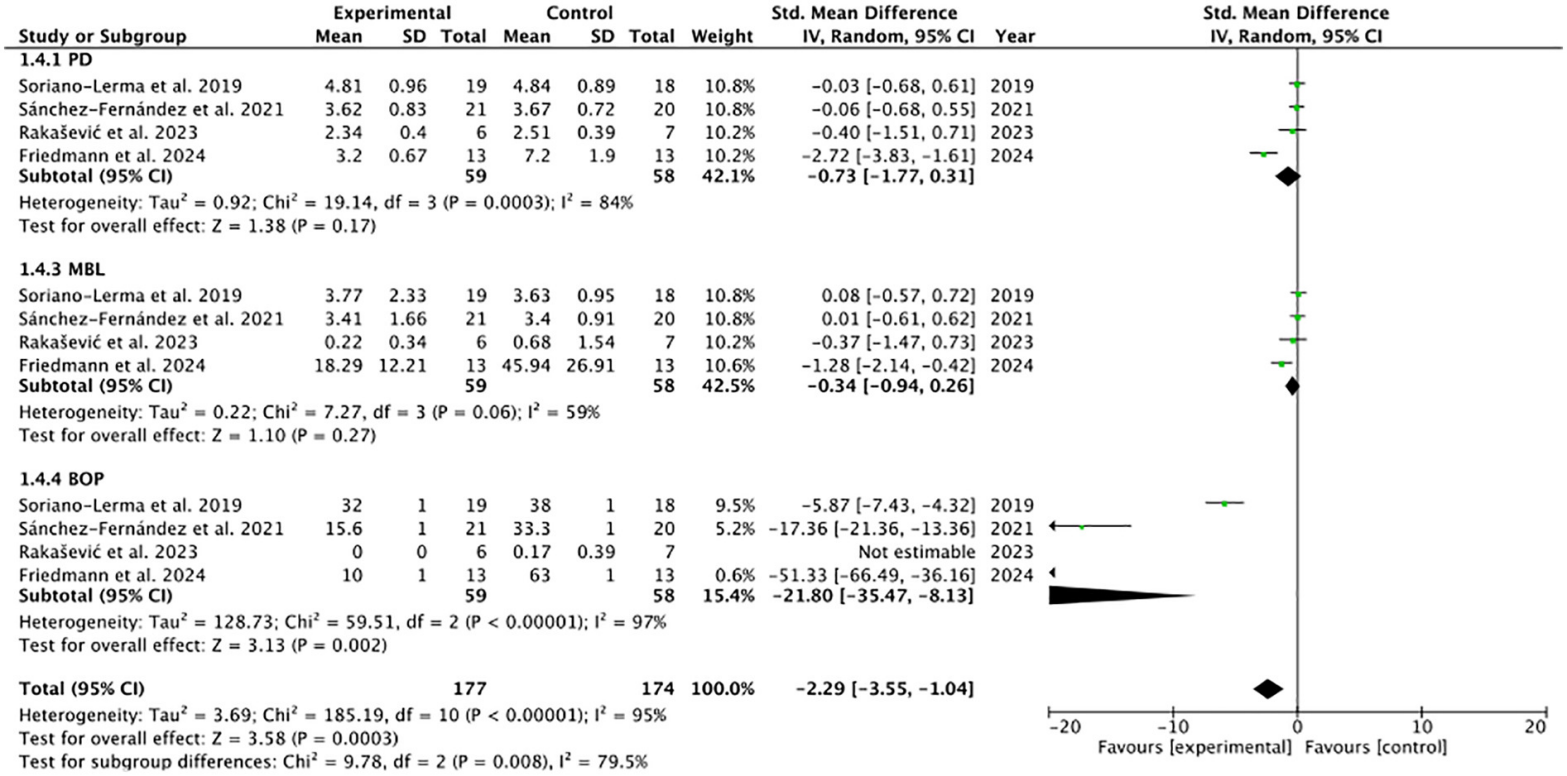
Forest plot of the pooled studies.

Separate individual meta-analyses were performed for each of the variables analysed (PD, AL, MBL and BOP). The meta-analysis for the BOP and PD subgroups showed the highest statistical significance (*p* < 0.00001 and *p* = 0.0003, respectively), with considerable heterogeneity (I^2^ = 97% and 84% respectively) and a trend towards significance for the MBL subgroup (*p* = 0.06), although with moderate heterogeneity (I^2^ = 59%) (Figures 2–5). Meta-analysis of pooled studies showed statistical significance towards the experimental group (*p* = 0.007) and substantial heterogeneity (I^2^ = 79.5%). Not included in this meta-analysis was the LA subgroup, which showed zero heterogeneity (I^2^ = 0%) and for which a separate fixed-effect meta-analysis was performed (Figure 6). No analysis of adverse effects was performed due to lack of dat.

**Figure 7:**
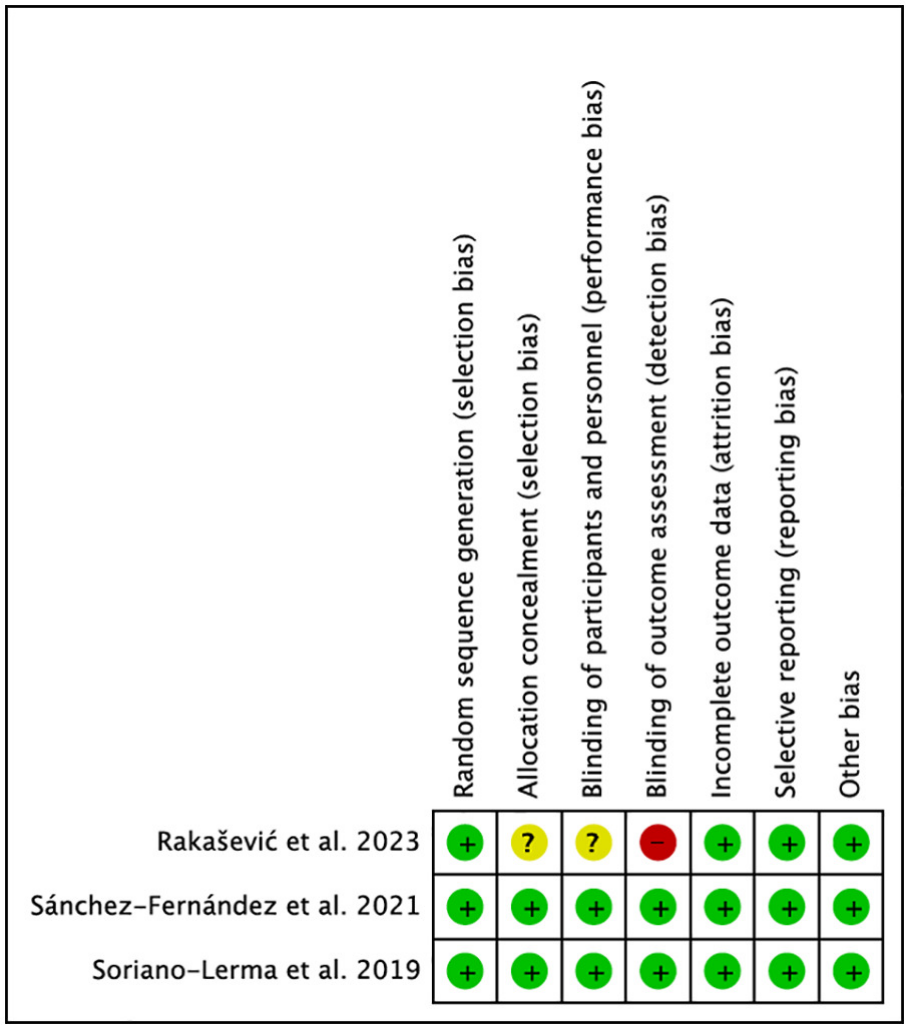
Risk of bias.

Risk of bias assessment is one of the pillars of evidence-based medicine; therefore, two reviewers (NL-V and AL-V) independently analysed the quality of included studies according to the Cochrane Risk of Bias tool. Two of the included studies (19, 20) met the criteria. The study by Rakašević et al. (21) had the highest number of biases, especially in the domains “Allocation concealment”, “Blinding of participants and staff” and “Blinding of outcome data” (Figure 7). The case-control study, for obvious reasons, could not be evaluated.

### 3.4 Publication bias

**Figure 8:**
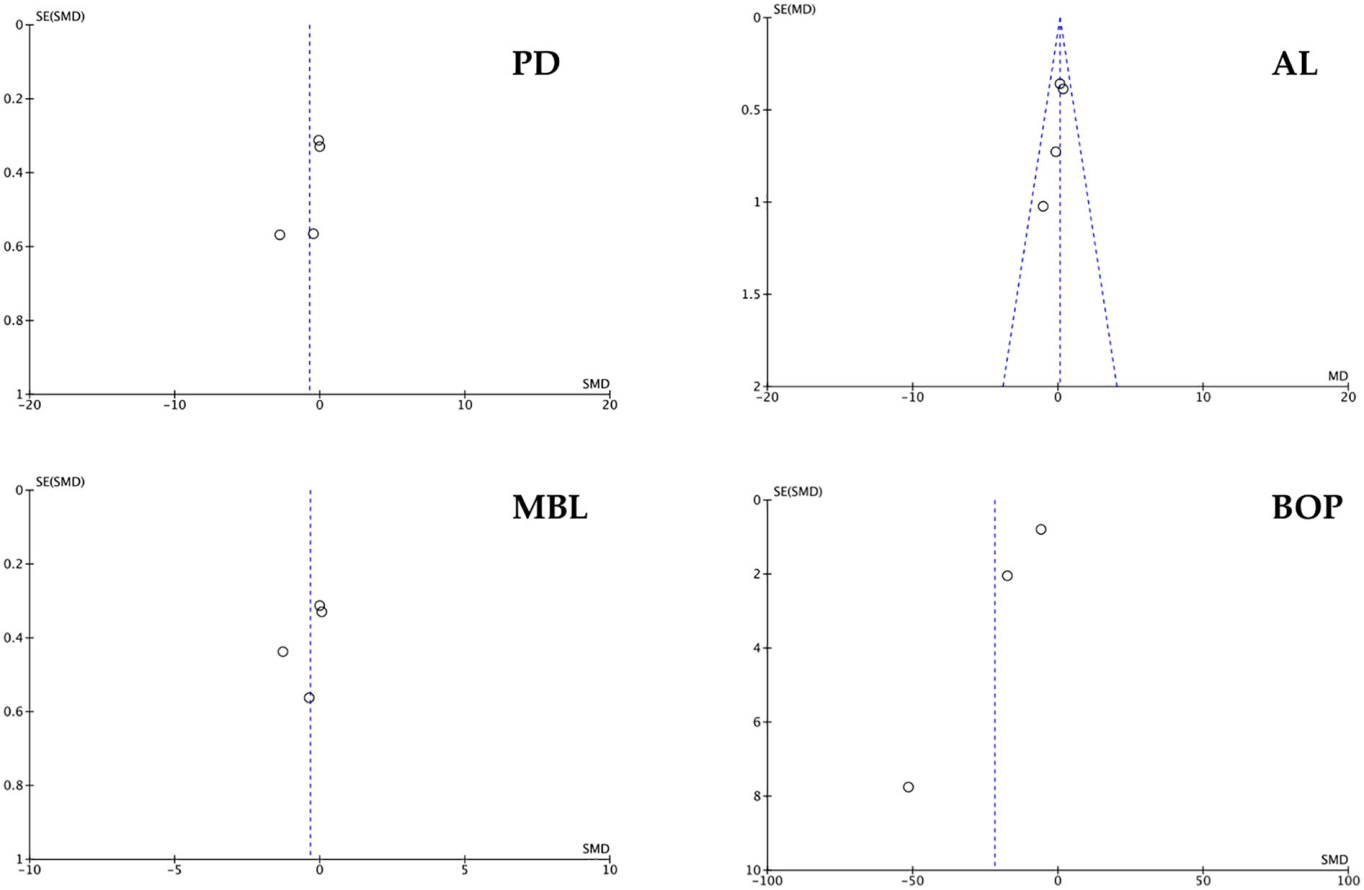
Funnel plot of PD, AL, MBL and BOP. For the AL parameter, a fixed effects funnel plot was performed.

**Figure 9:**
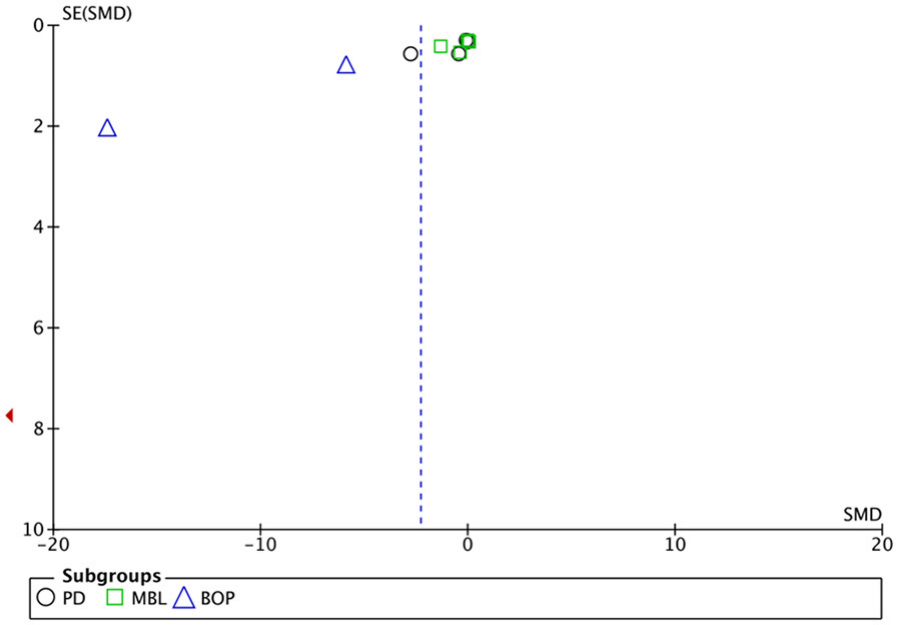
Funnel plot of clustered studies.

The graphs in Figures 8, 9 (except for AL), where the *x*-axis represents the observed results and the *y*-axis the standard error, show a large asymmetry, indicative of publication and reporting bias.

## 4 Discussion

Since so few RCTs have investigated the benefits of HA in peri-implant disease, we set out to prepare this systematic review with meta-analyses that would help to better understand the efficacy of the product in the pathogenesis of inflammatory peri-implant disease. Based on the best available evidence (i.e., three RCTs and one prospective study), we were unable to draw clear conclusions on its clinical efficacy.

Certain studies have highlighted that HA plays an important role in both periodontal repair and regeneration (25). In addition, non-cross-linked HA is considered to enhance tissue lubrication in cartilage, joints, and bones, guiding cell growth and differentiation, as well as accelerating the regeneration process and the healing and repair of chronic wounds (26). Its anti-inflammatory action in the healing process of hard and soft tissues has also been hypothesized (7, 27, 28).

A recent systematic review (29) showed that mechanical debridement alone or with complementary treatment, was not capable of eradicating the predominant pathogens from peri-implantitis sites and in this respect, certain *in vitro* studies have demonstrated the ability of HA to provoke a bacteriostatic response by reducing pathogenic periodontal bacteria and decreasing bacterial recolonization after mechanical debridement (30, 31). Subsequent to these studies, Soriano-Lerma et al. (19) demonstrated in an RCT on 63 subjects and 104 implants placed, the ability of HA to reduce early biofilm colonising bacteria (*Streptococcus*, *Veillonella* and *Rothia*), as well as a mild action on mid-colonisers (*Prevotella* and *Campylobacter*), and its ineffectiveness once late colonisers have established in the peri-implant area. They even reported that the use of HA in advanced stages of peri-implantitis produces a protective action of the peri-implant area against bacterial colonisation, considered the main aetiological cause of complications (32). Early colonisers provide a breeding ground for the colonisation of anaerobic periodontopathogenic bacteria (19), however, mid and late colonisers, with a Gram-negative anaerobic profile, are characteristic of periodontal diseases, although they have also been recognised in peri-implantitis (33, 34).

Inflammation of the peri-implant mucosa and the consequent bone loss are characteristic of peri-implantitis (35), and it has been shown that high molecular weight HA cancels out the immune response and prevents inflammation (36). In preclinical models it has been observed that supplementation with high molecular weight HA is associated with antiapoptotic, antioxidant and anti-inflammatory effects (37) and, in this sense, the study by Soriano-Lerma et al. and Sánchez Fernández et al. (19, 20) used high molecular weight al HA, which could have influenced the treatment results. However, other previous studies had demonstrated the beneficial effects of high molecular weight HA, one in a microbiome analysis (19) and another, a pilot study that evaluated the application of HA as a nebulizer (38). On the other hand, the molecular cross-linking of HA seems to improve its properties by achieving better bioavailability and resistance to degradation (39) and in this respect some research has resorted to cross-linked HA after subgingival instrumentation and has shown gains in clinical attachment levels and reductions in pocket depth levels (40). In our review, only one study (22) resorted to cross-linked HA as adjuvant therapy post-decontamination of the exposed implant surface.

In this context, reconstruction of bone defects caused by peri-implantitis has been proposed with the aim of limiting peri-implant mucosal recession and thus achieving bone regeneration around the implant (41). Since HA is attributed osteoinductive properties that would favor osteogenesis and bone regeneration through osteogenic cell stimulation and differentiation (in addition to influencing angiogenesis and bone neovascularization), it could play a role in this aspect (42). Rakašević et al. (21) in a RCT study on a sample of 13 patients and 19 sites with peri-implantitis, found significant values in terms of vertical bone gain of the marginal bone in the HA-treated group compared to the control (*p* < 0.05), as well as a significant improvement with respect to the absence of BOP. Friedmann et al. (22) also found a substantial reduction in the number of sites with BOP at 12-month follow-up (*p* < 0.0001). These results, in agreement with previous preclinical studies (43), could be due to the ability of HA to act as an anti-inflammatory agent by stopping the production of pro-inflammatory cells, however they disagree with the results obtained by Rakašević et al. (21) who only found a complete reduction of BOP in 20% of cases. Sánchez Fernández et al. (20) also observed a greater reduction in bleeding on probing in the experimental group, although without statistical significance (*p* = 0.07). However, a recent meta-analysis (44) showed that the prevalence of peri-implantitis was around one third in both BOP-positive implants and patients and cautioned that, although this is an indicative clinical factor in the diagnosis of peri-implantitis, clinicians should be aware of the significant false-positive rates of BOP. Other authors (45) have also commented that the value of BOP as a diagnostic tool for peri-implantitis would only fluctuate between 0% and 52% and before establishing a diagnosis of peri-implantitis, this parameter should be evaluated along with other parameters such as visual signs of inflammation, probing depth and progressive bone loss.

During tissue injury, HA is actively produced to regulate inflammatory cell activation and repair of injured tissues. All this results in an innate response that in turn regulates the behavior of epithelial cells and fibroblasts (46). For all these reasons and its anti-inflammatory, immunomodulatory, healing and tissue regenerative properties, HA is an attractive molecule for alternative treatments to conventional ones in pathologies of inflammatory origin (47).

Proinflammatory cytokines (IL-1β, TNF-α, IL-6, IL-17 and IL-12) play an important role in the initiation and progression of inflammatory diseases, and elevated crevicular concentrations of these proinflammatory biomarkers have been associated with peri-implantitis, especially IL-1β, which has been identified as a major contributor to bone loss, and elevated levels of IL-1 β in saliva are considered to reflect a local inflammatory response in peri-implant tissues (48, 49). Similarly, TNF-α is implicated in the destruction of peri-implant tissues by activating immune cells and in-creasing the production of other proinflammatory cytokines (50). Consistently, research has shown that increased levels of TNF-α in saliva are often associated with elevated levels of IL-1 β. These results suggest that the presence of elevated levels of IL-1 β and TNF-α in saliva may serve as a potential biomarker for the diagnosis and follow-up of peri-implantitis (51); however, Sanchez-Fernandez et al. (18) found that only those cases with a PD ≥5 mm showed a significantly greater reduction (*p* = 0.04) in IL-1β concentrations at 45 days, along with a greater reduction in BOP in the experimental group.

Oral hygiene has a very significant impact on bone stability around osseointegrated implants, and poor oral hygiene is considered to be related to greater bone loss (52). A retrospective cohort study on a predictive model of peri-implantitis that evaluated 254 implants, reported that the most influential factor in predicting survival was the time the implant had been functioning, followed by oral hygiene (53). Various studies have compared different products for the control of peri-implant health (54, 55). An interesting study by de Araujo et al. compared peri-implant health (hard and soft tissue) in 50 edentulous patients and a total of 120 implants, using HA gels or chlorhexidine in the patient maintenance protocol, reporting that both products improved gingival patterns, however, chlorhexidine did so with a constant level of dental plaque index or an increase in supragingival calculus, something that the group treated with HA did not show, and they suggested administering HA in the first 2 months and chlorhexidine between 2 and 6 months. In terms of bacteriology, they highlighted the bacteriostatic effect of HA on microorganisms such as *Porphyromonas gingivalis*, *Aggregatibacter actinomycetemcomitans* and *Staphylococcus aureus*, which leads to a reduction in the risk of postsurgical infection and promotes more predictable regeneration (56). In a similar vein, Soriano-Lerma et al. (19) reported on the ability of HA to reduce the bacteria that colonize the biofilm early on and that the use of HA in advanced stages of peri-implantitis produces a protective action in the peri-implant area against bacterial colonization.

Sánchez-Fernández et al. (20) evaluated the MBL in 104 implants, finding at 45 days a stability of the parameter in the experimental group and increased in the control group, which they attributed to the short duration of the follow-up period or to the fact that the product did not reach the bottom of the peri-implant pocket and, they therefore suggested a longer use, since in a previous study they had reported the opposite, that the application of high molecular weight HA in post-extraction sockets produced an increase in bone formation in the experimental group at 45 days (57). Rakašević et al. (21), after 6 months, also achieved successful treatment of bone defects caused by peri-implantitis, using a bovine bone substitute and HA, without additional MBL loss; however, the best results obtained in reference to this parameter, in the 15 implants analysed, were reported by Friedmann et al. (22) with more than 69% gain of mineralised tissue. Nevertheless, surgical debridement techniques have been used in the past. Used in the latter two studies could bias the excellent results obtained.

It is known that increased PD and AL, due to the loss of supporting bone, decreases the osseointegration of the coronal portion of the implant and, in addition, the rough and threaded surfaces of the implants make their decontamination difficult (58). In this aspect, Sánchez-Fernández et al. (20) used HA gel vs. placebo to reduce peri-implant pockets and increase attachment levels, obtaining values that only approached statistical significance (*p* = 0.06 for AL and *p* = 0.08 for PD). Rakašević et al. and Friedmann et al. (21, 22), used bone substitutes in combination with HA gel or HA-functionalised membranes, respectively, obtaining significant reductions in PD (3.9 ± 1.8 mm) compared to surgical treatment alone, which, according to recent meta-analyses, only obtained a mean reduction of 1.27 mm (59, 60).

Our study found in the analysis of pooled studies a strong statistical significance in favour of the intervention group over the control group (*p* = 0.00001), although heterogeneity was considerable (I^2^ = 93%). For this reason, we consider it a strength of our meta-analysis that the studies reviewed showed promising results for the use of HA in the treatment of peri-implant pathologies. However, more studies are needed to provide a more detailed understanding of the mechanisms of HA degradation in order to improve its biomedical applications and develop methods that allow for simple administration. It would also be necessary to study its mode of action in inflammatory pathologies, in order to make the most of this powerful molecule.

On the other hand, it should be noted that our systematic review and meta-analysis has a number of limitations and the results obtained should be taken with caution. First, we found significant heterogeneity among the studies that evaluated HA in the treatment of peri-implantitis (except for the AL parameter which yielded an I^2^ = 0%), due to study design, application of the product (alone or in combination with others), in combination with surgical or non-surgical treatments, doses and outcome assessment. Secondly, three of the included studies were RCTs and one was a prospective case series, and the risk of bias and methodological quality could not be assessed. Therefore, well-designed RCTs with long-term follow-up periods justifying the benefits of HA in the treatment of peri-implantitis are justifiable and necessary. Finally, it would be necessary to test and evaluate HA protocols and formulations suitable for clinical applications.

## 5 Conclusions

Within these limitations, current data indicate that the application of HA, alone or in combination with other materials in bone defects, may provide additional clinical benefits when used as an adjunct to surgical and non-surgical periodontal treatment. In addition, topical application of HA in peri-implantitis appears to reduce inflammation. However, due to the high risk of bias and heterogeneity, well-designed RCTs evaluating the role of this material in various clinical scenarios are needed.

## Data Availability

The original contributions presented in the study are included in the article/Supplementary Material, further inquiries can be directed to the corresponding author.
